# TERRA beyond cancer: the biology of telomeric repeat‐containing RNAs in somatic and germ cells

**DOI:** 10.3389/fragi.2023.1224225

**Published:** 2023-08-10

**Authors:** Julieta Rivosecchi, Emilio Cusanelli

**Affiliations:** Laboratory of Cell Biology and Molecular Genetics, Department of Cellular, Computational and Integrative Biology—CIBIO, University of Trento, Trento, Italy

**Keywords:** TERRA, telomeres, telomerase, fibroblasts, stem cells, post-mitotic cells, germ cells

## Abstract

The telomeric noncoding RNA TERRA is a key component of telomeres and it is widely expressed in normal as well as cancer cells. In the last 15 years, several publications have shed light on the role of TERRA in telomere homeostasis and cell survival in cancer cells. However, only few studies have investigated the regulation or the functions of TERRA in normal tissues. A better understanding of the biology of TERRA in non-cancer cells may provide unexpected insights into how these lncRNAs are transcribed and operate in cells, and their potential role in physiological processes, such as aging, age-related pathologies, inflammatory processes and human genetic diseases. In this review we aim to discuss the findings that have advanced our understanding of the biology of TERRA using non-cancer mammalian cells as a model system.

## Introduction

Eukaryotic chromosomes have evolved specialized nucleoprotein structures termed telomeres to protect their extremities. Mammalian telomeres are constituted by telomeric DNA, telomere-binding proteins forming the shelterin protein complex, and telomeric repeat-containing RNAs, known as TERRA (de Lange, 2009). The telomeric DNA is an array of repetitive sequences that enables the recruitment of telomere binding proteins which mediate telomere homeostasis. In vertebrates, the telomeric DNA sequence consists of TTAGGG repeats (Moyzis et al., 1988) extending for 9–15 kb in humans (de Lange et al., 1990). In several organisms the actual end of telomeres contains a 3′ G-rich overhang that invades the upstream double-strand telomeric region, forming the t-loop structure (Griffith et al., 1999; Doksani et al., 2013).

In mammals, both the double-stranded and the single-stranded telomeric DNA sequences are bound by the shelterin complex which is essential for telomere function and structure. A key role of the shelterin complex is to solve the “end protection problem” that arises from the necessity of cells to discriminate the end of chromosomes from the extremities of DNA double strand breaks (DSBs), occurring due to exogenous and endogenous insults (de Lange, 2009; 2018). DSBs are quickly sensed by the DNA damage response (DDR) that triggers a cell cycle arrest and activation of DNA repair mechanisms (Harper and Elledge, 2007). The absence of shelterin proteins or unwinding of the t-loop leads to telomere dysfunction, activation of DDR pathways at chromosome ends and consequent cell cycle arrest (Sfeir and de Lange, 2012; Doksani et al., 2013). In the absence of functional check-points, instigation of DDR signaling at telomeres results in the activation of DNA repair processes with consequent deleterious outcomes, including chromosome end-to-end fusions and genomic instability (Denchi and de Lange, 2007; Sfeir and de Lange, 2012; Doksani et al., 2013; Ly et al., 2018).

Due to the inability of the DNA replication machinery to fully duplicate the extremities of linear chromosomes (Watson, 1972; Olovnikov, 1973), in the absence of maintenance mechanisms telomeres erode during each cell division, a process known as the “end replication problem” (Allsopp et al., 1992). In human cells, telomere erosion ultimately triggers an irreversible cell cycle arrest known as replicative senescence that limits the replicative capacity of cells (Smith and Pereira-Smith, 1996; Braig et al., 2005). Telomere attrition can be counteracted by the addition of telomeric repeats to the 3′ overhang of chromosomes via the ribonucleoprotein telomerase complex (Kim et al., 1994). The essential components of telomerase are the catalytic subunit telomerase reverse transcriptase (TERT) and the RNA subunit telomerase RNA component (TERC or TR) (Greider and Blackburn, 1985; Greider and Blackburn, 1989). In adult tissues, telomerase activity can be detected in stem cells (Wright et al., 1996; Cong et al., 2002). Furthermore, approximately 90% of human cancers upregulate telomerase to overcome replicative senescence and attain proliferative immortality (Kim et al., 1994). In humans, telomerase is also active during early embryogenesis while it is inactivated by the silencing of TERT in somatic cells from early development (Wright et al., 1996). Thus, in most human tissues, telomeres shorten with age (Demanelis et al., 2020). Furthermore, senescent cells with damaged telomeres accumulate during aging in various organisms (Krishnamurthy et al., 2004; Herbig et al., 2006; Jeyapalan et al., 2007). Persistent activation of DDR pathways at dysfunctional chromosome ends has been observed in aging post-mitotic cells, including neurons and adipocytes, also independently of telomere length (Fumagalli et al., 2012; Hewitt et al., 2012). In line with these findings, telomere dysfunction is considered an important contributor to organismal aging (Rossiello et al., 2022; López-Otín et al., 2023).

Increasing evidence indicates that telomere homeostasis is regulated by the telomeric repeat-containing RNA TERRA, long noncoding RNAs transcribed from chromosome ends that associate with telomeres (Feuerhahn et al., 2010). TERRA functions at chromosome ends by base-pairing with telomeric repeats, forming RNA:DNA hybrids (R-loops), and by interacting with telomere binding proteins to regulate telomeric chromatin, telomeric DNA replication, telomere mobility, telomere protection and telomere elongation (Flynn et al., 2011; de Silanes et al., 2014; Azzalin and Lingner, 2015; Chu et al., 2017a; Niehrs and Luke, 2020). TERRA also acts in *trans*, regulating gene expression as well as participating in extranuclear processes (Chu et al., 2017a; Bettin et al., 2019). Most studies on TERRA have been performed using cancer cell lines. These studies indicated TERRA as a key player of telomere biology and genome integrity (Cusanelli and Chartrand, 2015). Furthermore, TERRA deregulation was found to impact cancer cell survival pointing to TERRA as an attractive therapeutic target (Azzalin and Lingner, 2015). In addition to these findings, TERRA expression has been reported in normal mammalian cells, including fibroblasts, embryonic stem cells and germ cells (Azzalin et al., 2007; Schoeftner and Blasco, 2008; Reig-Viader et al., 2013; Reig-Viader et al., 2014b; de Silanes et al., 2014; Sagie et al., 2014; Chu et al., 2017a; Kordowitzki et al., 2020). Furthermore, TERRA has been detected in human post-mitotic cells (Diman et al., 2016). Studying TERRA in non-cancer settings will be important to help delineate its involvement in genetic pathologies, as well as physiological processes including aging and in age-related diseases (Maicher et al., 2012; Aguado et al., 2020).

In this minireview, we first provide an overview of the current knowledge on TERRA biogenesis in mammalian cells. We then discuss the main findings on the regulation and functions of TERRA obtained in non-cancer cells. In accordance with the topic of this special issue, we lastly report and discuss the evidence on TERRA biology in mammalian germ cells.

## Overview of TERRA biogenesis

**FIGURE 1:**
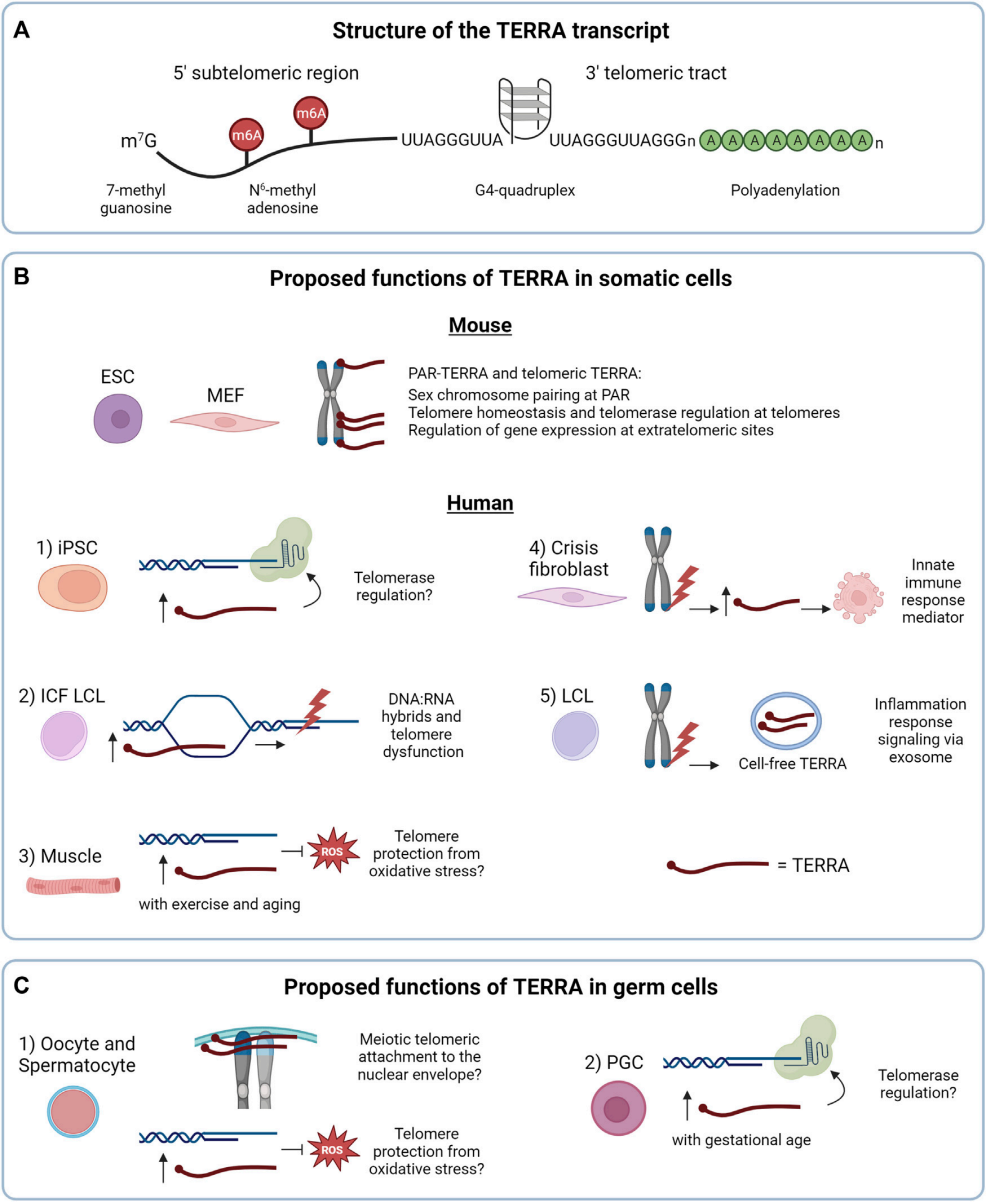
**(A)** Structure of the TERRA transcript. **(B)** Proposed functions of TERRA in somatic cells. In mouse cells the nuclear TERRA population is composed of TERRA expressed from pseudoautosomal regions (PAR-TERRA) and telomeric TERRA. Both RNA species are depicted in red. As shown in mouse embryonic stem cells (mESC), mouse embryonic fibroblast (MEF) and other mouse cell lines, TERRA binds to PAR, telomeres and extratelomeric genes, likely exerting different functions. In human cells, 1) TERRA may regulate telomerase activity in induced pluripotent stem cells (iPSC), a function shared with mouse iPSCs. 2) In lymphoblastoid cell lines (LCL) from patients with the immunodeficiency, centromeric instability and facial anomalies (ICF) syndrome high TERRA levels lead to accumulation of telomeric RNA:DNA hybrids that could result in telomeric dysfunction. 3) Under endurance exercise or aging, TERRA may protect telomeres from reactive oxygen species (ROS) in skeletal muscle. 4, 5) Regarding the extranuclear functions of TERRA: 4) in crisis fibroblasts, TERRA transcribed from dysfunctional telomeres may activate innate immune responses and induce cell death, as a tumor-suppressor mechanism. 5) In LCL, TERRA transcribed from dysfunctional telomeres is secreted as cell-free TERRA into exosomes to the extracellular environment to activate an inflammatory response. **(C)** Proposed functions of TERRA in germ cells. 1) TERRA could participate in meiotic processes and/or protect telomeres from oxidative stress, as proposed in human and mouse oocytes and spermatocytes. 2) In mouse primordial germ cells (PGC), TERRA may regulate telomerase activity. In **(B,C)** TERRA molecules are shown in red.

In mammalian cells, TERRA molecules are generated by RNA polymerase II from the telomeric C-rich strand, with transcription starting from subtelomeres and proceeding toward chromosome ends (Azzalin et al., 2007; Schoeftner and Blasco, 2008). TERRA transcripts contain a 5′ sequence derived from subtelomeres, followed by tracts of telomeric repeats at their 3′ end (UUAGGG in vertebrates) (Azzalin et al., 2007; Schoeftner and Blasco, 2008; Porro et al., 2010; Diman and Decottignies, 2018) (Figure 1A). TERRA molecules range from 100 nt to 9 kb in length in mammals, and contain a canonical 7-methylguanosine (m^7^G) 5′ cap (Porro et al., 2010). 7% of the total human TERRA population presents a 3′ polyadenylated tail (Figure 1A), which determines the stability and localization of the transcripts (Azzalin and Lingner, 2008; Schoeftner and Blasco, 2008; Porro et al., 2010). While the mechanism of TERRA polyadenylation remains to be elucidated, it has been recently observed in different cancer cell lines that polyadenylation occurs on TERRA transcripts expressed from specific telomeres, indicating that this process can be regulated in a telomere-specific manner (Savoca et al., 2023). Indeed, the hnRNP RALY and the poly(A) binding protein PABPN1 interact with TERRA in HeLa cells and regulate its stability through mechanisms that are defined by the presence of the poly(A) tail. A previous study supported the role of other hnRNPs, such as hnRNP F, in TERRA stability in mouse cells (de Silanes et al., 2014). Which RNA decay enzymes target TERRA in human and mouse cells remains to be defined. Interestingly, emerging evidence indicates that not only the 3′ end of TERRA but also its subtelomeric sequences are important for the stability of the transcripts. Indeed, it has been recently reported that the methyltransferase METTL3 catalyzes the N6-methyladenosine (m6A) modification of TERRA transcripts (Chen et al., 2022) (Figure 1A). These modifications occur within the subtelomeric sequences of TERRA and are recognized by the m6A reader YTHDC1 which stabilizes TERRA molecules. METTL3 or YTHDC1 depletion promotes TERRA degradation (Chen et al., 2022) in human cancer cells using alternative telomere lengthening mechanisms (ALT) which do not involve telomerase activity (Bryan et al., 1995). Further studies will be required to elucidate the mechanisms of TERRA posttranscriptional modifications and the processes involved in TERRA transcripts decay in non-cancer cells. These processes may well be influenced by the structure of these RNAs.

Indeed, the G-rich telomeric tract of TERRA can form stable G-quadruplex (G4) structures composed by the stacking of planar arrangements of four guanines held together by hydrogen bonds (Randall and Griffith, 2009; Collie et al., 2010; Xu et al., 2010) (Figure 1A). G4 structures of TERRA were proposed to mediate the interaction with proteins involved in telomere stability (Takahama et al., 2013; Wang et al., 2017; Mei et al., 2021).

TERRA transcription in mammals is tightly regulated by multiple mechanisms. A subset of human subtelomeres contain CpG-island promoters located upstream of TERRA transcription start sites (Nergadze et al., 2009; Porro et al., 2014a). Methylation of CpG-rich TERRA promoters is regulated by DNMT1 and DNMT3b DNA methyltransferases and represses TERRA expression (Nergadze et al., 2009; Feretzaki et al., 2019; Le Berre et al., 2019). The chromatin state of subtelomeres and telomeres is also a major determinant for the regulation of TERRA transcription. Large amount of evidence supports a model in which a decrease in repressive chromatin marks at these genomic regions leads to chromatin relaxation and increased levels of TERRA (Schoeftner and Blasco, 2008; Marion et al., 2009; Arnoult et al., 2012; Episkopou et al., 2014; Rippe and Luke, 2015; Diman and Decottignies, 2018; Barral and Déjardin, 2020; Novo, 2021). However, a study by Gauchier et al. (2019) in mouse embryonic stem cells (mESCs) suggests that the heterochromatin mark H3K9me3 at telomeres catalyzed by the histone methyltransferase SETDB1 positively correlates with TERRA levels (Gauchier et al., 2019). Differences in H3K9me3 density at subtelomeres versus telomeres and the use of different cell lines may explain the discrepancy of the results between studies (Barral and Déjardin, 2020; Udroiu and Sgura, 2020; Novo, 2021). Finally, TERRA levels are cell cycle regulated. TERRA accumulates in G1 phase, prior to the replication of telomeres, and decreases in S phase in telomerase-positive cells (Porro et al., 2010; Flynn et al., 2011; Arnoult et al., 2012; Flynn et al., 2015). The nature of the cell cycle control of TERRA levels remains to be elucidated.

Several studies indicate that different cell types express different TERRA levels and, importantly, TERRA levels markedly vary among telomeres of a given cell type suggesting that chromosome ends regulate TERRA expression by distinct mechanisms. The study of the telomere-specific regulation of TERRA is fundamental to understanding how the multiple cellular functions of this telomeric lncRNA are coordinated in cells (Rivosecchi J., Jurikova K., Cusanelli E., submitted for publication).

## The biology of TERRA in non-cancer cells

### Expression and proposed functions of TERRA in somatic cells

Investigations of TERRA expression in mammalian non-tumor settings have provided several unexpected findings that critically contributed to our understanding of TERRA biology. Using a CHIRT protocol developed from the combination of ChiRP (Chromatin isolation by RNA Purification) and CHART (Capture Hybridization Analysis of RNA Targets), Chu and others investigated the genomic binding sites of TERRA transcripts in mESCs (Chu et al., 2017a). Surprisingly, the authors observed that while TERRA associates with telomeres, most of its binding sites map within extratelomeric regions and do not overlap with telomere-like sequences (Figure 1B). Furthermore, the authors used antisense oligonucleotides (ASO) to downregulate TERRA transcripts through RNase H-mediated degradation. Depletion of TERRA in mESCs resulted in deregulation of hundreds of genes containing, or mapping nearby, TERRA binding sites. This study provided the first evidence supporting the role of TERRA in gene expression regulation (Figure 1B). A subsequent work in mESCs has shown that TERRA recruits PRC2 to differentiation genes to control their expression in a TRF1-dependent manner (Marión et al., 2019), further supporting a role of TERRA at extratelomeric sites.

The biology of TERRA in mice has revealed distinctive features compared to humans. In early studies, direct visualization of TERRA molecules by RNA fluorescence *in situ* hybridization (RNA FISH) in mouse mammary epithelial cells and immortalized mouse embryonic fibroblasts (iMEF) revealed that TERRA transcripts accumulate close to the inactive X chromosome (Schoeftner and Blasco, 2008). This pattern was in stark contrast with the localization of TERRA in human cells, forming discrete foci detected throughout the nucleus, a subset of which localizes at telomeres (Azzalin et al., 2007; Schoeftner and Blasco, 2008). A subsequent study reported that in mESCs TERRA localizes at both sex chromosomes, a pattern that changed during differentiation, when TERRA transcripts predominantly localized to the heterochromatic sex chromosome (Zhang et al., 2009). This conundrum was, at least in part, later solved by the identification of the pseudoautosomal regions (PAR) of the sex chromosomes as the main loci transcribing telomeric repeat-containing RNAs in normal and cancer mouse cells (Chu et al., 2017b; Viceconte et al., 2021). Also in this study, CHIRT-seq experiments confirmed that these RNAs, termed PAR-TERRA, associate with numerous extratelomeric sites throughout the genome and at chromosome ends. Intriguingly, it was proposed that PAR-TERRA mediates homologous sex chromosome pairing in female and male mESCs (Chu et al., 2017b) (Figure 1B). Notably, low levels of TERRA were detected also from chromosome ends in mESCs and mouse induced pluripotent stem cells (iPSCs) (Toubiana et al., 2020; Viceconte et al., 2021). Furthermore, a previous study suggested that in MEF and murine iPSCs TERRA is expressed mainly from chromosome 18 (de Silanes et al., 2014). Expression of TERRA from this chromosome end was confirmed in other studies, although in these works telomere 18 TERRA was found to be expressed at similar levels compared to TERRA transcripts generated from other telomeres (Toubiana et al., 2020; Viceconte et al., 2021). Discrepancies between these studies could be attributed to the different assays used for TERRA detection, and in the mouse cell models analyzed, differentiated versus undifferentiated cells or immortalized fibroblasts versus embryonic stem cells. The telomeric origin of mouse TERRA is still in need of a unified consensus (Diman and Decottignies, 2018). A better understanding of the mechanisms regulating TERRA expression in mouse will help solve this matter.

The telomeric functions of TERRA in mouse cells remain to be dissected. TERRA depletion in mESCs resulted in an increased number of chromosome ends displaying DDR activation and telomere instability, as observed by the detection of telomere duplications, fusions and telomere free ends, suggesting that downregulation of TERRA may lead to telomere dysfunction (Chu et al., 2017a). Several telomeric roles have been proposed for TERRA in human cancer cells, where TERRA molecules can regulate the chromatin state of chromosome ends (Deng et al., 2009; Arnoult et al., 2012; Montero et al., 2018), assist fork restart upon replicative stress (Beishline et al., 2017), promote telomere capping *in vitro* (Flynn et al., 2011), act as scaffold molecule to facilitate the recruitment of proteins and enzymes to chromosome ends impacting on DDR activation (Porro et al., 2014a; Porro et al., 2014b), and promote homologous recombination through R-loops formation (Arora et al., 2014; Silva et al., 2019; Feretzaki et al., 2020; Silva et al., 2021; Kaminski et al., 2022; Yadav et al., 2022). Several reviews have examined TERRA functions in human cancer cells during recent years (Azzalin and Lingner, 2015; Bettin et al., 2019; Domingues-Silva et al., 2019; Lalonde and Chartrand, 2020; Bhargava et al., 2022).

Interestingly, downregulation of TERRA in mESCs resulted in increased telomerase activity as detected by TRAP assay (Chu et al., 2017a). These findings are in line with *in vitro* evidence indicating that TERRA-mimicking oligonucleotides inhibit telomerase activity from human and mouse cellular extracts (Schoeftner and Blasco, 2008; Redon et al., 2010). However, how TERRA regulates telomerase *in vivo* in mammalian cells remains to be defined. Indeed, TERRA has been shown to interact with both TERT and TR in human cells (Redon et al., 2010). Yet, the mechanism of TERRA-mediated telomerase regulation may be highly controlled in cells, and evidence obtained also in non-cancer cells indicates that TERRA levels can positively correlate with telomerase activity. In this regard, different studies reported increased TERRA levels during cellular reprogramming of human neonatal foreskin fibroblasts or MEF to iPSCs (Marion et al., 2009; Yehezkel et al., 2011; de Silanes et al., 2014; Toubiana et al., 2020), processes that lead to telomerase activation and telomere elongation (Marion et al., 2009; Yehezkel et al., 2011) (Figure 1B-1). Notably, these findings are in line with the capability of telomerase to elongate an engineered chromosome end over-expressing TERRA from an inducible promoter in HeLa cells (Farnung et al., 2012). Thus, further studies will need to be performed to elucidate the function of TERRA in telomerase regulation in mammalian cells (Lalonde and Chartrand, 2020).

Investigations of non-cancer somatic cells have also provided insights into the regulation of TERRA expression and highlighted the potential involvement of TERRA in genetic diseases. In this regard, studies in primary cells from patients with the immunodeficiency, centromere instability and facial anomalies (ICF) syndrome, a rare autosomal disorder caused by mutations in the gene DNMT3b, represent a striking example of the impact of subtelomeric methylation on TERRA regulation (Toubiana and Selig, 2020). Hypomethylated subtelomeres in ICF human fibroblasts, lymphoblastoid cells and iPSCs result in high TERRA levels (Yehezkel et al., 2008; Deng et al., 2010; Yehezkel et al., 2013; Sagie et al., 2017; Toubiana et al., 2019). Conversely, loss of DNMT3b in mouse cells does not result in increased TERRA levels, possibly due to the paucity of CpG regions at mouse subtelomeres (Diman and Decottignies, 2018; Toubiana et al., 2020). Interestingly, human ICF cells show increased DNA:RNA hybrids formation associated with elevated TERRA levels, that lead to replication stress and telomeric DNA damage (Figure 1B-2). These processes could contribute to the accelerated telomere shortening and premature senescence observed in these cells (Sagie et al., 2017). These studies have provided key insights into the transcriptional regulation of TERRA and on the characterization of telomeric hybrids and their impact on telomere integrity in non-cancer cells, also unveiling the potential involvement of TERRA in a rare genetic disease.

Intriguingly, mechanisms of TERRA expression regulation have been studied also in human primary tissues and postmitotic cells. In particular, RNA FISH combined with immunofluorescence (IF) enabled the detection of TERRA at telomeres in human muscle biopsies (Diman et al., 2016). RT-qPCR analyses of TERRA levels in these tissue samples revealed upregulation of TERRA mediated by the antioxidant transcription factor NRF1 during endurance exercise. In addition to these findings, this study reported that TERRA transcripts localize to telomeres in human myotubes differentiated from myoblasts, in which TERRA levels are also regulated by NRF1, promoting TERRA expression under oxidative stress. As discussed by the authors, these results suggest that TERRA may be part of an antioxidant response in skeletal muscle cells to counteract exercise-induced oxidative stress (Diman et al., 2016). This function may be exerted by TERRA also in aged muscle tissues (Balan et al., 2021) (Figure 1B-3).

Emerging evidence using human fibroblasts indicates that TERRA can also exert extranuclear functions mediating the activation of the innate immune response upon telomere shortening. Indeed, (Nassour et al., 2023) demonstrated that TERRA generated from dysfunctional telomeres is upregulated in checkpoint-deficient fibroblasts that undergo crisis after bypassing senescence. By performing RNA immunoprecipitation (RIP) experiments using total cellular extracts from crisis fibroblasts, the authors showed that TERRA associates with the cytosolic innate immunity sensor Z-DNA binding protein 1 (ZBP1) that in turn activates the mitochondrial antiviral-signaling protein (MAVS) and induces cell death (Nassour et al., 2023) (Figure 1B-4). This work suggests that TERRA can shuttle from the nucleus to the cytoplasm and opens new avenues to the study of extranuclear functions of TERRA. The mechanism regulating the nuclear export of TERRA transcripts remains to be identified. Furthermore, TERRA could be also present in the cytoplasm embedded in membranous vesicles as previously suggested in human cancer and lymphoblastoid cell lines (LCLs) (Wang et al., 2015; Wang and Lieberman, 2016), although these RNAs mainly correspond to short TERRA species consisting of telomeric repeat sequences (Figure 1B-5). In this context, TERRA-containing vesicles released to the extracellular environment from cells with dysfunctional telomeres were suggested to elicit an inflammatory response by immune cells, supporting not only extranuclear but also extracellular functions of TERRA in humans (Wang and Lieberman, 2016). Further studies on TERRA subcellular localization and the identification of the RNA-binding proteins that regulate the nuclear export of TERRA will help clarify the mechanisms of the nuclear-cytoplasm trafficking of TERRA molecules and provide additional insights into their extranuclear functions.

### Expression and proposed functions of TERRA in germ cells

Only few studies on TERRA in germline cells are available in the literature most likely due to the complexity to obtain and isolate cells undergoing gametogenesis, especially female oocytes. In germ cells, telomeres not only protect chromosome ends, but they also participate in meiosis. Alterations of telomeric structure or telomere length can affect the formation of gametes (Reig-Viader et al., 2016; Tardat and Déjardin, 2018; Rossiello et al., 2022). While the role of TERRA in gametogenesis has not been fully elucidated yet, several pieces of evidence indicate that TERRA expression may have an impact in this process. By studying human ovarian samples from fetuses of 22 gestational weeks, Reig-Viader et al. (2013) detected TERRA transcripts in prophase I primary oocytes using RNA FISH. By combining TERRA RNA FISH with TRF2 IF the authors observed that most TERRA foci co-localized with telomeres (Reig-Viader et al., 2013). Furthermore, a subsequent study from the same group reported the detection of TERRA foci in prophase I spermatocytes and about half of them colocalizing with telomeres (Reig-Viader et al., 2014b). These findings suggest that during meiotic prophase I TERRA transcripts are expressed and localize to telomeres. During gametogenesis, telomeres are known to tether to the nuclear envelope, facilitating the alignment, pairing, synapsis and recombination of homologous chromosomes (Klutstein and Cooper, 2014; Reig-Viader et al., 2016). It is intriguing to hypothesize that TERRA molecules may participate in some of these processes (Figure 1C-1), considering their proposed roles in homologous sex chromosome pairing in mESCs and MEFs (Chu et al., 2017b) and in homologous recombination process at telomeres in human ALT cancer cells (Silva et al., 2021; 2022).

Interestingly, RNA FISH/IF experiments have shown that a number of TERRA foci colocalize with TERT in germ cells, independently of the gender (Reig-Viader et al., 2013; Reig-Viader et al., 2014b). These colocalization events correlate with an inactive state of telomerase during meiosis (Reig-Viader et al., 2013; Reig-Viader et al., 2014b; Reig-Viader et al., 2016). Whether TERRA association may represent a mechanism of telomerase inhibition in germ cells is still unknown.

A new work in mouse oocytes and spermatocytes provided the first evidence of the presence of polyadenylated TERRA and PAR-TERRA in germ cells (Biswas et al., 2023). The authors used RT-qPCR and RNA FISH to show that in spermatocytes defective for the meiotic cohesin SMC1β subunit, TERRA levels are upregulated and correlate with telomeric RNA:DNA hybrids formation, detected by IF using the S9.6 antibody, and with open subtelomeric chromatin. Moreover, SMC1β deficiency impacts TERRA polyadenylation pattern, which is telomere-specific, and correlates with increased TERRA localization at damaged telomeres (Biswas et al., 2023). Interestingly, Smc1β^−/−^ mouse are infertile and most of telomeres in Smc1β^−/−^ spermatocytes and oocytes fail to attach to the nuclear envelope during prophase I (Adelfalk et al., 2009). The authors argue that loss of the meiotic cohesin SMC1β may impact sister chromatid cohesion but also produce TERRA-associated telomere defects, both contributing to age-related aneuploidy in oocytes, supporting the model in which TERRA plays a role during gametogenesis. Intriguingly, in human cancer cells, the cohesin component Rad21 and the chromatin organizing factor CTCF bind to TERRA promoters and control TERRA transcription (Deng et al., 2012). Similar to germ cells, depletion of CTCF and cohesin result in telomere damage in cancer cells, but unlike Smc1β^−/−^ spermatocytes, TERRA levels are downregulated upon depletion of the cohesin component Rad21. These findings suggest that the control of TERRA levels by cohesin is important to maintain telomere integrity in mice as in humans, although the mechanisms of TERRA regulation by cohesin may be dissimilar.

TERRA transcripts were detected by RNA FISH and RT-qPCR also in mouse primordial germ cells (PGCs), the embryonic precursors of germ cell lineages, prior to meiosis. In these cells, TERRA foci number per cell and TERRA transcripts levels increase with gestational age, with female PGCs showing higher TERRA foci compared to male (Brieño-Enríquez et al., 2019), suggesting that TERRA is regulated in a gestational and gender manner in these cells. Interestingly, the increase in the number of TERRA foci at late gestational stage coincides with the global DNA demethylation that occurs in gonads (Seisenberger et al., 2012). As mice subtelomeric regions do not contain canonical CpG promoters (Toubiana et al., 2020), this effect may indirectly regulate TERRA transcription or stability. Interestingly, also in this cellular context TERRA foci colocalize with TERT (Brieño-Enríquez et al., 2019). Notably, the increase in TERRA levels with gestational age correlates with a decrease in TERT expression (Brieño-Enríquez et al., 2019). Whether TERRA molecules regulate TERT in PGCs remains to be determined (Figure 1C-2).

Germ cells, and in particular oocytes arrested in meiotic prophase I experience chronic oxidative stress (Reig-Viader et al., 2016; Kordowitzki, 2021; Derevyanko et al., 2022). Several studies using somatic cells of different origin have shown that TERRA levels increase during cellular stress, upon DNA damage at telomeres (Porro et al., 2014a), heat shock (Schoeftner and Blasco, 2008; Koskas et al., 2017), as well as chemotherapy treatment or serum starvation (Tutton et al., 2016) in cancer cells, and upon oxidative stress in normal cells (Diman et al., 2016; Galigniana et al., 2020). As mentioned above, it was proposed that TERRA transcripts participate in a telomeric antioxidant response triggered by oxidative stress (Diman et al., 2016). Indeed, telomeric DNA is sensitive to oxidative damage due to its high content in guanines, which are oxidized to 8-oxo guanines (8-oxoG) (Oikawa and Kawanishi, 1999). UUAGGG repeats oxidation of TERRA molecules may shield TTAGGG repeats of chromosome ends from reactive oxygen species (ROS) (Diman et al., 2016). Thus, it is tempting to hypothesize that TERRA may also have a role in preventing telomeric DNA damage and telomere loss in germ cells (Figure 1C-1). However, in this regard, a recent study from Kordowitzki et al. (2020) showed that oocytes from young mice (6 months old) present similar number of TERRA foci compared to oocytes from older mice (24 months of age) (Kordowitzki et al., 2020). Furthermore, the number of TERRA foci during the first divisions of the embryos was not affected by the age of oocyte donors upon oocyte fertilization (Kordowitzki et al., 2020). It will be interesting to investigate whether the localization of TERRA at telomeres is influenced by the age of the oocytes and the oxidation state of TERRA molecules in these cells. Further studies on the mechanisms regulating TERRA expression and localization in aging germ cells are required to elucidate the role of TERRA in protecting telomeres in oocytes exposed to aging-associated DNA damage.

TERRA detection in germ cells has been mostly studied by RNA FISH experiments. RT-qPCR analyses have been performed in some instances using primer pairs to assess TERRA levels from specific telomeres. While RNA FISH allows the detection of TERRA in single cells, confirming TERRA expression and providing key information on its localization and extent of transcripts clustering, the number of TERRA foci detected by this technique will depend on the experimental settings (e.g., RNA FISH protocol, microscope used), making it difficult to compare different studies. Furthermore, the number of TERRA foci will be influenced by the length of the 3′ end of TERRA molecules, which base pairs with the fluorescent probe, and by the extent of clustering of the transcripts. RNase treatment controls are also useful to rule out the possibility that single strand DNA may be detected. These limitations considered, single cell analyses represent a key approach to study RNAs and to directly visualize transcripts at single cell resolution in fixed samples.

Indeed, the subcellular localization of TERRA transcripts may be determinant for instructing the alternative functions proposed for TERRA in non-cancer cells. As discussed above, TERRA plays a wide variety of roles that are dependent on the cellular context, the expression levels and localization pattern.

## Concluding remarks

The study of TERRA in non-tumor human and mouse cells is contributing to advance our understanding of the mechanisms regulating TERRA expression as well as of its telomeric and extratelomeric functions (Table 1).

**TABLE 1:** TERRA detection in non-cancer somatic and germ cells.

Organism	Cells	Technique	Telomeric RNA species	Reference
Mouse	iMEFs and mammary epithelium	RNA FISH	TERRA	Schoeftner and Blasco (2008)
Mouse	pMEFs and ESCs	RNA FISH, Northern blot	TERRA	Zhang et al. (2009)
Mouse	MEFs, ESCs and iPSCs	Northern blot, RNA Dot blot	TERRA	Marion et al. (2009)
Mouse	pMEFs and iPSCs	RNA FISH, Northern blot, RT-qPCR, RNA-seq	TERRA	de Silanes et al. (2014)
Mouse	ESCs	RNA FISH, Northern blot, RT-PCR, RNA-seq, CHIRT	PAR-TERRA and telomeric TERRA	Chu et al. (2017b), Chu et al. (2017a)
Mouse	MEFs, ESCs and immortalized cell lines	RNA FISH, RNA Dot blot, RT-qPCR	PAR-TERRA and telomeric TERRA	Viceconte et al. (2021)
Mouse	MEFs, ESCs and iPSCs	Northern blot, RT-qPCR	TERRA	Toubiana et al. (2020)
Mouse	Primordial germ cells	RNA FISH, RT-qPCR	TERRA	Brieño-Enríquez et al. (2019)
Mouse	Oocytes and spermatocytes	RNA FISH, RT-qPCR	PAR-TERRA and telomeric TERRA	Biswas et al. (2023)
Mouse and bovine	Oocytes and early embryos	RNA FISH	TERRA	Kordowitzki et al. (2020)
Human and mouse	Oocytes and spermatocytes	RNA FISH, RT-PCR	TERRA	Reig-Viader et al. (2013), Reig-Viader et al. (2014b)
Human	iPSCs	Northern blot, RT-PCR	TERRA	Yehezkel et al. (2011)
Human	ICF LCLs	RNA Dot blot	TERRA	Deng et al. (2010)
Human	ICF iPSCs, LCLs and fibroblasts	Northern blot, RT-qPCR	TERRA	Yehezkel et al. (2008), Yehezkel et al. (2013), Sagie et al. (2014), Sagie et al. (2017), Toubiana et al. (2019)
Human	Skeletal muscle and myoblasts	RNA FISH, RT-qPCR	TERRA	Diman et al. (2016), Balan et al. (2021)
Human	LCLs and immortalized fibroblasts	RNA FISH, Northern blot, RNA Dot blot, RT-qPCR, RNA-seq	Cell-free TERRA	Wang et al. (2015), Wang and Lieberman (2016)
Human	p53 and RB-deficient fibroblasts under crisis	RNA Dot blot, RT-qPCR	TERRA	Nassour et al. (2023)
Human	Spermatocytes from infertile males	RNA FISH	TERRA	Reig-Viader et al. (2014a)

i/pMEF, immortalized/primary mouse embryonic fibroblasts; ESCs, embryonic stem cells; iPSCs, induced pluripotent stem cells; ICF, immunodeficiency, centromeric instability, facial anomalies; LCLs, lymphoblastoid cell lines; PAR, pseudoautosomal regions; CHIRT, combination of ChIRP (Chromatin Isolation by RNA, Purification) and CHART (Capture Hybridization Analysis of RNA, Targets).

Furthermore, several lines of evidence indicate the involvement of TERRA transcripts in the telomere biology of ICF patients derived cells, at least in part through the formation of telomeric RNA:DNA hybrids. Intriguingly, altered metabolism of RNA:DNA hybrids structures has been described in cells from patients carrying other genetic diseases, including amyotrophic lateral sclerosis 4 (ALS4) (Grunseich et al., 2018), ataxia oculomotor apraxia type 2 (AOA2) (Becherel et al., 2015; García-Muse and Aguilera, 2019) and Aicardi–Goutières syndrome (AGS) (Lim et al., 2015). It will be interesting to investigate whether in these pathological conditions impaired regulation of R-loops structures occurs also at telomeres, suggesting an involvement of TERRA in the diseases.

The expression of TERRA in germline cells during meiosis opens the intriguing possibility that this RNA may participate in the meiotic process during gametogenesis. Alterations of telomere structure and shortening of telomeres in germ cells are associated with reduced fertility (Lopes et al., 2019; Rossiello et al., 2022) due to disrupted gametogenesis which in turn produces aneuploid gametes and miscarriages (Reig-Viader et al., 2016). It would be interesting to assess TERRA levels in these conditions to study its potential contribution to dysfunctional gametogenesis. In this regard, spermatocytes from infertile males have shown reduced number of TERRA foci and altered nuclear distribution compared to control individuals (Reig-Viader et al., 2014a), supporting the protective role that TERRA may exert at telomeres in germ cells.

Studying TERRA in mammalian models different from cancer cell lines will enable the investigation of new potential roles of these telomeric transcripts in more physiological conditions. Despite the differences in telomere biology between mice and humans, which include longer telomeres and broader expression of telomerase in mouse cells, and the distinct features of TERRA transcription regulation and genomic origins between the two species, mouse models may represent an important tool to decipher the physiological functions of TERRA during embryogenesis and in differentiated somatic cells. Generating TERRA KO models will be a difficult task given to the multiple genomic sites transcribing telomeric repeat-containing RNAs at chromosome ends and PARs. Nevertheless, the study of TERRA in the available conditional knock-out models for TERRA-interacting factors or telomere-binding proteins may help advancing our understanding on TERRA biology and to verify the potential involvement of TERRA in the phenotypes of these mice (Cheong et al., 2003; Li et al., 2019; Gaspar et al., 2022). These studies are expected to have implications for the study of TERRA biology also in humans. Indeed, several TERRA-binding proteins are conserved between mice and humans, such as the BLM helicase and the paraspeckles component NONO, a condition that could be attributed to the same G-rich telomeric sequence of TERRA in these species (Deng et al., 2009; Flynn et al., 2011; Petti et al., 2019; Viceconte et al., 2021). Along these lines, studies of TERRA in non-tumor settings will have implications also for cancer research. Cancer arises as a consequence of mutations occurring in normal cells that disrupt oncogenes and oncosuppressor genes, leading to their transformation into malignant cells (Vogelstein and Kinzler, 2015). Organisms have developed tumor suppressive mechanisms to counteract DNA mutations and cellular transformation, most of which remain to be defined (Hanahan and Weinberg, 2011). Studies conducted in normal cells can thus enable the identification of these pathways. Oxidative stress has been identified as one of the multiple factors stimulating tumorigenesis (Hayes et al., 2020). In this regard, the proposed function of TERRA in counteracting oxidative stress in skeletal muscle cells and aged muscle tissues (Diman et al., 2016; Balan et al., 2021) highlights that telomere transcription may participate in a tumor suppressive pathway to prevent oxidative damage to genomic DNA and consequent accumulation of mutations, also in aged tissues in which DNA damage accumulates (Schumacher et al., 2021). Direct evidence for this hypothesis will need to be provided.

Therefore, it will be interesting to study TERRA in aging organisms and tissues. Indeed, cancer is considered a disease of aging (The importance of aging in cancer research, 2022) and several hallmarks of aging overlap with the hallmark of cancer, including genomic instability and DNA damage (Hanahan and Weinberg, 2011; López-Otín et al., 2023). Furthermore, recent evidence indicates that aging tissues contribute to the formation of a tumor promoting microenvironment, sustaining cancer cell malignancy and resistance to therapy. This condition is instructed for a good part by normal cells surrounding the tumor creating a proinflammatory and tumor-permissive environment (Fane and Weeraratna, 2020; Lex et al., 2020). The aging process is associated with accumulation of DNA damage (Pereira and Ferreira, 2013; López-Otín et al., 2023), a condition which results in upregulation of TERRA in different cellular settings (Porro et al., 2014a; Porro et al., 2014b; Tutton et al., 2016). The fact that TERRA-containing exosomes stimulate a proinflammatory response in peripheral blood mononuclear cells suggests that extracellular function of TERRA may participate in the formation of a tumor-permissive environment (Wang et al., 2015). Thus, studies of TERRA in aging organisms and tissues may help verify this hypothesis.

TERRA may also play a cell autonomous role in promoting cellular transformation. Indeed, increased expression of TERRA from dysfunctional telomeres has been shown to contribute to DDR activation at chromosome ends (Porro et al., 2014a; Porro et al., 2014b), a condition well known to fuel genome instability (O’Sullivan and Karlseder, 2010). Human precancerous cells accumulate DDR as result of oncogene activation (Bartkova et al., 2005; Gorgoulis et al., 2005). It will be interesting to study whether this condition results in TERRA deregulation and consequent telomere dysfunction. One could speculate that during aging increased TERRA levels in normal and/or precancerous cells, may induce cell autonomous effects by sustaining telomere dysfunction as well as non-cell autonomous consequences by promoting a pro-inflammatory microenvironment, both activities may seed cancer development. For this reason, studying the mechanisms of TERRA transcription, processing and localization in non-tumor cells may be instrumental to dissect the functions of these telomeric transcripts in cancer.

The use of yeast as a model system has been critical to study the biology of TERRA and telomeric R-loops, also during senescence (Coulon and Vaurs, 2020; Niehrs and Luke, 2020; Zeinoun et al., 2023). Expanding our analyses to different eukaryotes, such as plants (Vrbsky et al., 2010; Majerová et al., 2011), protozoa (Morea et al., 2021; Saha et al., 2021), and zebrafish (Park et al., 2019; Idilli et al., 2020; El Maï et al., 2023) will enable us to broaden our knowledge on this telomeric RNAs potentially unveiling novel and conserved functions. Importantly, species-specific mechanisms regulating TERRA expression will most likely surface, as it has been observed for the different impact of DNA methylation in TERRA expression between mice and humans (Toubiana et al., 2020). Furthermore, it has been reported that TERRA may act as a positive regulator of telomerase in the budding yeast *S. cerevisiae* and the fission yeast *S. pombe* (Cusanelli et al., 2013; Moravec et al., 2016); conversely TERRA may repress telomerase in human cells (Redon et al., 2010). Thus, species-specific TERRA functions most likely exist. Studying TERRA in multiple model systems will enable us to help unveil unexpected and intriguing findings which lie ahead on the biology of this much studied and yet not completely understood RNA.
